# Dataset of taxonomic identification from surface water in the coastal area of Peninsular Malaysia

**DOI:** 10.1016/j.dib.2024.110683

**Published:** 2024-06-26

**Authors:** Ayu Nabila Khairul Anuar, Nur Khayyirah Muhammad Zamri, Muhammad Abid Mohd Yuzaidey, Suriyanti Su Nyun Pau, Nur Ili Hamizah Mustaffa

**Affiliations:** a Department of Environment, Faculty of Forestry and Environment, Universiti Putra Malaysia, 46400 Serdang, Selangor, Malaysia; b Department of Earth Sciences and Environment, Faculty of Science and Technology, Universiti Kebangsaan Malaysia, Selangor, Malaysia

**Keywords:** Phytoplankton, Diatom, Cell density, Southwest monsoon, Northeast monsoon

## Abstract

This article describes the abundance of phytoplankton community structures in Port Dickson, Negeri Sembilan and Pulau Tinggi, Johor during the Southwest and Northeast Monsoons and includes data from 48 selected sampling sites collected between July and December 2023. The seawater samples from 1-meter depth were obtained by using a Niskin water sampler, concentrated in a 50 ml centrifuge tube and immediately preserved with Lugol's iodine solution. The data include phytoplankton density (cell L^−1^), the total density of phytoplankton in each station, and the total number of genera obtained in every station. Additional data are presented, including chlorophyll-a concentration, as a proxy for biomass and photosynthetic active radiation. This article presents data on 30 genera, including unidentified genera, as well as the percentage of the main community group.

Specifications Table

**Table:** 

Subject	Oceanography, Marine Biology
Specific subject area	Taxonomic identification of phytoplankton species at Port Dickson, Negeri Sembilan, and Pulau Tinggi, Johor
Type of data	Table
Data collection	An inverted microscope (Olympus x70) was utilised for phytoplankton counting and genus identification. The ambient photosynthetic active radiation (PAR) was measured using a PAR Smart Sensor (S-LIA-M003, Onset HOBOware). Chlorophyll-a concentration was determined by filtering 800 – 1000 mL of the sample onto GF/F (Whatman), extracted with 90 % acetone for 12 h in the dark at 4 °C, centrifuged for 10 min, and then measured with a UV–visible spectrophotometer (UVD-3000, Labomed Inc. USA). The chlorophyll-a concentration was calculated according to [1], substituting the equation from [2].
Data source location	Seawater samples were collected at two different coastal areas of Peninsular Malaysia: Pulau Tinggi, Johor (2°17′60.00″ N, 104° 06′60.00″ E) and Port Dickson, Negeri Sembilan (2° 32′ 13.85″ N, 101° 48′ 20.56″ E), Malaysia. Data are stored at Universiti Putra Malaysia
Data accessibility	Raw data are presented in the articles. Repository name: Mendeley Data [3] Data identification number: DOI: 10.17632/rw9b982xh9.2 Direct URL to data: https://doi.org/10.17632/rw9b982xh9.2
Related research article	Not related

## 1 Value of the Data

• The samples were obtained from two locations: Port Dickson, Negeri Sembilan (west coast) and Pulau Tinggi, Johor (east coast). Data from Port Dickson were collected during the Southwest (SW) and Northeast (NE) monsoons.
• Data can serve as a point of reference for the analysis of phytoplankton variability in the coastal area and continental shelf of Peninsular Malaysia.
• Data can be used to explain the potential sources of surface-active substances and biogenic volatile organic compounds emissions from the surface ocean.
• A comparative study of the phytoplankton population in Port Dickson and Pulau Tinggi can be conducted from these datasets.

## 2 Background

Phytoplankton are valuable indicators of shifting oceanographic conditions, climate change, and declining water quality because of their high turnover rates and sensitivity to environmental changes [4]. We provide a dataset for the spatial variation of phytoplankton in Port Dickson (west coast) and Pulau Tinggi (east coast), Malaysia during the SW and NE monsoons. In Malaysia, there are still limited studies on the phytoplankton's taxonomic and biomass, particularly for both monsoon events. Previous studies show that phytoplankton production can produce a significant amount of surface-active substances (SASs) [5,6] and biogenic volatile organic compounds (BVOCs) emissions from the surface ocean [[7], [8], [9]]. This research hypothesizes that phytoplankton biomass will be higher during the SW monsoon than the NE monsoon. The datasets are available under the FAIR principle of Findability, Accessibility, Interoperability, and Reusability.

## 3 Data Description

**Fig. 1:**
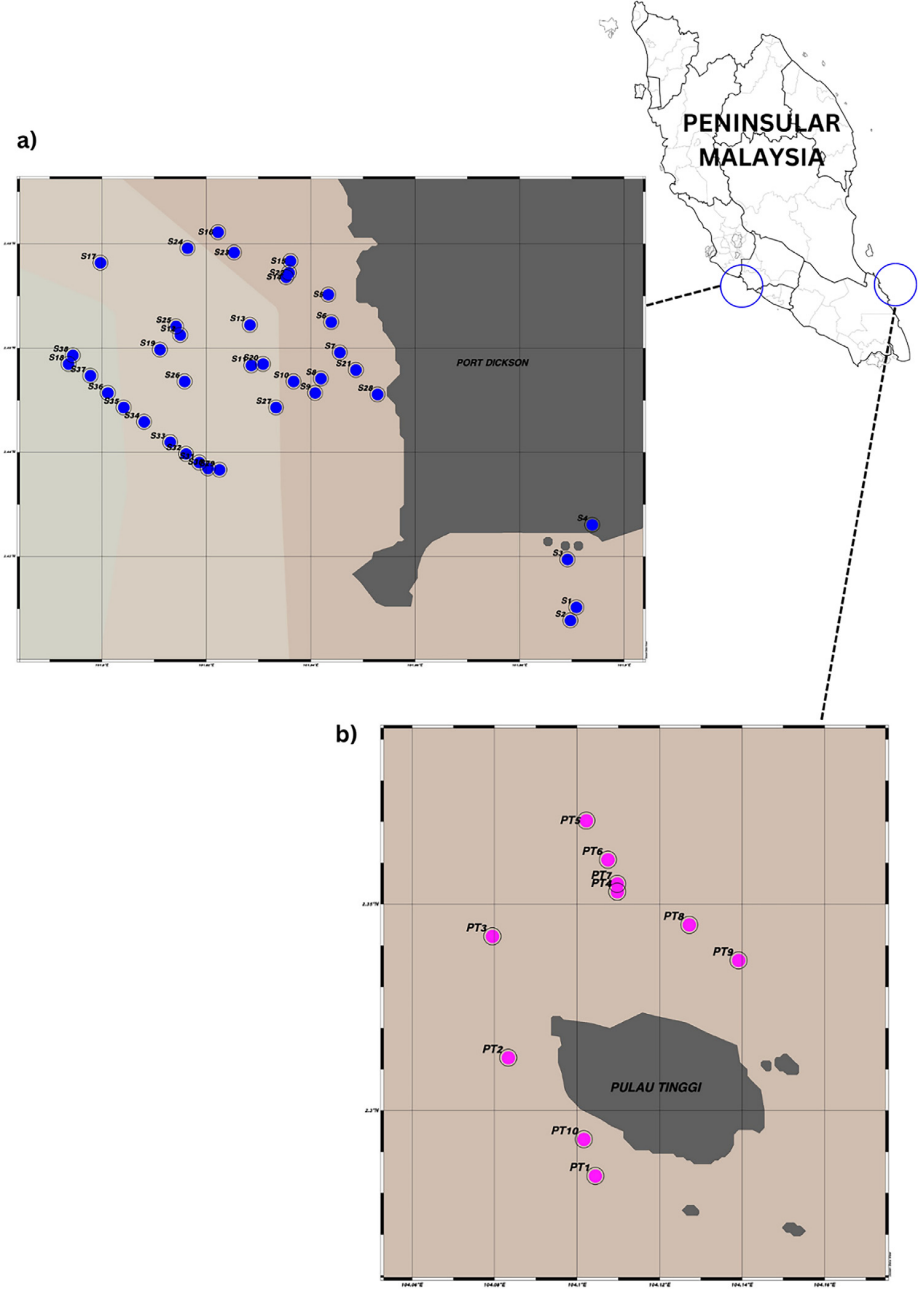
Map of the sampling locations at a) Port Dickson, Negeri Sembilan and b) Pulau Tinggi, Johor.

The data was collected at Port Dickson and Pulau Tinggi, Malaysia during the SW and NE monsoons (Table 1 and Fig. 1). The datasets involve information on phytoplankton density consisting of 30 genera from 48 stations, including unidentified genus. The information on chlorophyll-a concentration and PAR values were also presented in Table 1, meanwhile, Table 2, Table 3, Table 4 provide information on phytoplankton density (cell L^−1^), total number of genera, and total density of phytoplankton. Table 5, Table 6 show the percentage abundances for the major groups of phytoplankton in both locations.

**Table 1:** Location of sampling stations, PAR values and Chl-a concentrations.

Date	Stations	Local time	Longitude (°E)	Latitude (°N)	PAR (µmol/m^2^/s)	Chl-a (µg/L)
11/08/2023	PT1	16:37:00	104.29556	2.29111	475	1.40
	PT2	17:10:00	104.23250	2.42667	314	0.96
	PT3	17:48:00	104.19500	2.42250	147	0.60

12/08/2023	PT4	09:44:00	104.34694	2.53139	1436	0.57
	PT5	09:59:00	104.27222	2.55250	1072	0.56
	PT6	10:14:00	104.17472	2.45694	1269	0.60
	PT7	10:31:00	104.19639	2.10556	1217	0.76
	PT8	10:57:00	104.37083	2.44889	1269	0.70
	PT9	11:21:00	104.33944	2.51306	1405	0.79
	PT10	11:49:00	104.12944	2.44722	1423	0.68

27/7/2023	S1	11:48:11	101.89083	2.41028	1623	1.02
	S2	11:58:18	101.88972	2.40778	1848	0.70
	S3	12:18:31	101.88917	2.41944	1748	0.88
	S4	12:38:44	101.89389	2.42611	1723	1.11

20/09/2023	S5	10:00:00	101.84333	2.47028	1029	1.03
	S6	10:26:00	101.83417	2.46500	881	0.94
	S7	10:42:00	101.84556	2.45917	1440	2.16
	S8	11:00:00	101.84194	2.45417	1109	2.47
	S9	11:20:00	101.84082	2.45146	1530	0.70
	S10	11:30:00	101.83667	2.45361	1692	1.95
	S11	11:50:00	101.82879	2.45757	1910	1.05
	S12	12:05:00	101.81500	2.46250	1931	1.07
	S13	12:21:00	101.82833	2.46444	1289	0.75
	S14	12:35:00	101.83523	2.46738	1413	0.46

26/10/2023	S15	09:52:57	101.83611	2.47667	>2500	1.21
	S16	10:18:09	101.82222	2.48222	>2500	0.71
	S17	11:27:50	101.79972	2.47639	>2500	0.66
	S18	11:37:55	101.79361	2.45694	>2500	0.60
	S19	11:58:05	101.81111	2.45972	>2500	0.64
	S20	12:18:15	101.83083	2.45694	>2500	0.73
	S21	12:48:30	101.84861	2.45583	>2500	0.63

23/11/2023	S22	10:46:13	101.83583	2.47444	821	1.12
	S23	11:06:23	101.82528	2.47833	881	0.99
	S24	11:26:33	101.81639	2.47917	739	0.82
	S25	11:51:45	101.81417	2.46417	471	0.91
	S26	12:06:52	101.83583	2.47444	481	0.83
	S27	12:27:02	101.83333	2.44861	674	0.81
	S28	12:52:14	101.85278	2.45111	964	0.65

28/12/2023	S29	10:41:06	101.82250	2.43667	1391	1.79
	S30	11:06:24	101.82028	2.43694	1131	0.80
	S31	11:21:35	101.81861	2.43806	1208	0.77
	S32	11:39:00	101.81611	2.43972	1368	0.83
	S33	11:56:00	101.81306	2.44194	1430	0.81
	S34	12:16:00	101.80806	2.44583	1112	0.89
	S35	12:29:00	101.80417	2.44861	709	1.41
	S36	12:39:00	101.80111	2.45139	521	1.60
	S37	12:50:00	101.79778	2.45472	799	0.78
	S38	13:01:00	101.79444	2.45861	1187	1.14

**Table 2:** Data on the density of phytoplankton species (cell L^−1^) and total no. of genus in Pulau Tinggi, Johor. Note: (-) no organisms found are described.

Genus	Stations									
	PT1	PT2	PT3	PT4	PT5	PT6	PT7	PT8	PT9	PT10
*Actinoptychus*	–	50	50	–	–	–	–	–	–	–
*Alexandrium*	–	–	–	–	–	–	50	–	–	–
*Bacteriastrum*	100	50	–	50	–	–	50	–	–	–
*Cerataulina*	50	–	–	–	–	–	–	–	–	–
*Ceratium*	–	–	–	–	–	–	–	–	50	–
*Chaetoceros*	–	50	50	–	–	50	50	50	50	–
*Coscinodiscus*	250	100	–	–	50	50	100	–	–	–
*Detonula*	–	–	50	–	–	–	–		–	–
*Diploneis*	–	–	–	–	–	–	–	50	–	–
*Ditylum*	50	50	50	–	–	–	–	50	50	–
*Guinardia*	100	–	50	150	–	50	–	–	50	–
*Hemiaulus*	500	100	–	50	–	50	–	–	50	–
*Lauderia*	200	–	–	–	50	50	50	50	–	–
*Leptocylindrus*	100	–	–	–	–	–	–	–	–	–
*Odontella*	50	50	50	–	–	50	–	50	50	–
*Planktoniella*	100	100	–	–	–	50	–	–	–	–
*Pleurosigma*	50	–	50	–	–	–	100	50	–	50
*Proboscia*	200	–	–	–	200	–	50	50	–	50
*Pseudo-nitzia*	50	250	50	–	50	–	–	–	–	–
*Rhizoselenia*	50	–	–	–	50	–	50	–	–	–
*Thalassionema*	50	50	–	–	–	–	–	–	–	–
*Thallasiosira*	–	–	–	–	–	–	–	–	–	–
*Triceratium*	50	–	50	50	–	–	–	–	–	–
*Tropidoneis*	–	–	–	–	–	–	50	–	50	–
**Total density of phytoplankton**	**1755**	**850**	**450**	**300**	**400**	**350**	**550**	**350**	**300**	**100**
**Total no of genus**	**16**	**10**	**9**	**4**	**5**	**7**	**9**	**7**	**7**	**2**

**Table 3:** Data on the density of phytoplankton species (cell L^−1^) and total no. of genus in during the Southwest monsoon Port Dickson, Negeri Sembilan. Note: (-) no organisms found are described.

Genus	Stations																				
	SW																				
	S1	S2	S3	S4	S5	S6	S7	S8	S9	S10	S11	S12	S13	S14	S15	S16	S17	S18	S19	S20	S21
*Alexandrium*	183	150	–	–	–	–	117	133	100	33	50	–	–	–	–	–	–	–	–	–	–
*Bacteriastrum*	50	–	–	17	–	–	–	–	–	–	–	–	–	–	–	–	–	–	–	–	–
*Ceratium*	–	–	–	–	33	33	–	–	–	–	–	–	–	–	13	–	–	–	–	–	13
*Chaetoceros*	3117	19,650	15,533	683	50	–	–	17	–	–	–	–	–	–	25	13	–	–	–	25	–
*Coscinodiscus*	100	450	250	150	83	183	50	13	100	–	100	83	67	83	13	25	13	13	25	–	13
*Ditylum*	83	350	200	50	33	–	50	17	–	–	–	33	–	–	–	–	–	–	–	–	–
*Diploneis*	–	–	–	–	–	–	–	–	–	50	–	–	–	–	–	13	–	–	–	–	–
*Leptocylindrus*	–	–	–	–	–	–	–	33	50	–	–	33	–	33	–	–	–	–	–	–	–
*Guinardia*	617	800	767	33	–	83	17	50	33	33	50	–	–	–	–	–	–	–	–	–	–
*Hemiaulus*	–	–	–	50	–	–	–	–	–	–	–	–	–	–	–	–	–	–	–	–	–
*Navicula*	–	–	–	–	–	–	–	–	–	–	–	–	–	–	–	–	–	–	–	–	–
*Lauderia*	*283*	550	267	183	–	–	17	–	–	–	–	–	–	–	25	–	25	13	13	25	25
*Leptocylindrus*	*–*	–	–	–	–	–	–	–	–	–	–	–	–	–	–	–	–	–	–	–	–
*Lithodesmium*	*–*	–	–	–	–	–	–	–	–	–	–	–	–	–	–	–	–	–	–	–	–
*Odontella*	50	83	33	67	50	–	–	–	50	–	50	33	50	50	13	25	13	13	13	25	–
*Proboscia*	617	17	–	–	50	–	–	33	50	–	–	–	–	–	–	13	–	25	25	13	13
*Pseudo-nitzia*	*–*	–	933	717	117	–	33	17	50	–	–	50	83	117	13	–	13	–	–	–	13
*Planktoniella*	*–*	17	50	33	–	–	–	–	–	–	–	–	–	–	–	–	–	–	–	–	–
*Pleurosigma*	*–*	67	17	–	33	–	–	33	–	–	50	33	50	33	13	–	–	–	13	–	–
*Protoperidinium*	*–*	–	17	–	–	–	–	–	–	–	–	–	–	–	–	–	–	–	–	–	–
*Pyrocystis*	–	17	–	–	–	–	–	–	–	–	–	–	–	–	–	–	–	–	–	–	–
*Pyrodinium*	50	–	–	–	–	–	–	–	–	–	–	–	–	–	–	–	–	–	–	–	–
*Stephanophycis*	–	50	–	–	–	–	–	–	–	–	–	–	–	–	–	–	–	–	–	–	–
*Thalassionema*	50	–	–	–	33	–	–	–	–	–	–	–	–	–	–	–	–	–	–	–	–
*Thallassiosira*	50	133	733	117	–	–	–	–	–	–	–	–	–	–	–	–	–	–	–	–	–
*Triceratium*	–	–	–	–	–	–	–	17	–	–	–	–	–	–	–	–	–	–	–	–	–
*Rhizoselenia*	33	–	50	50	–	–	–	–	–	50	–	–	–	–	–	13	13	13	–	25	–
*unknown*	–	–	–	–	–	–	–	–	–	–	–	–	–	–	–	–	–	–	–	–	–
Total density of phytoplankton	**5283**	**22,334**	**18,850**	**2150**	**199**	**299**	**167**	**363**	**433**	**133**	**250**	**265**	**250**	**316**	**115**	**102**	**77**	**77**	**77**	**113**	**77**
Total no of genus	**13**	**13**	**12**	**12**	**9**	**3**	**6**	**10**	**7**	**4**	**4**	**6**	**4**	**5**	**7**	**6**	**5**	**5**	**5**	**5**	**5**

**Table 4:** Data on the density of phytoplankton species (cell L^−1^ and total no. of genus) during the Northeast monsoon in Port Dickson, Negeri Sembilan. Note: (-) no organisms found are described.

Genus	Stations																
	NE																
	S22	S23	S24	S25	S26	S27	S28	S29	S30	S31	S32	S33	S34	S35	S36	S37	S38
*Alexandrium*	*–*	–	–	–	–	–	–	–	–	–	–	–	–	–	–	–	–
*Bacteriastrum*	*–*	–	–	–	–	–	–	–	–	–	–	–	–	–	–	–	–
*Ceratium*	*–*	–	–	–	–	–	–	–	–	–	–	–	–	–	–	–	–
*Chaetoceros*	*–*	–	–	50	–	–	–	–	–	–	–	–	–	–	–	–	–
*Coscinodiscus*	50	33	50	50	33	50	50	–	50	50	–	33	33	33	67	83	–
*Ditylum*	50	50	33	167	50	67	50	50	–	50	–	50	33	–	–	–	–
*Diploneis*	*–*	–	–	–	50	–	–	–	–	–	67	–	50	–	–	–	–
*Leptocylindrus*	*–*	–	–	33	100	–	–	–	–	–	–	–	–	–	–	–	–
*Guinardia*	33	–	–	–	–	50	–	–	–	–	–	–	–	–	33	67	–
*Navicula*	*–*	–	–	–	–	–	–	–	–	–	–	–	50	–	–	–	–
*Lauderia*	50	33	–	–	–	–	–	–	–	–	–	–	–	–	–	–	–
*Lithodesmium*	*–*	–	–	–	–	–	–	–	–	–	–	–	–	–	–	–	–
*Odontella*	*–*	–	50	50	–	–	–	50	–	17	33	–	33	–	33	33	–
*Proboscia*	50	–	–	83	–	–	33	–	–	50	–	33	83	–	–	–	–
*Pseudo-nitzia*	33	–	50	83	–	–	–	–	–	–	–	50	–	–	–	83	–
*Planktoniella*	*–*	–	–	–	–	–	–	–	–	–	–	–	–	–	–	–	–
*Pleurosigma*	50	–	67	–	33	33	–	67	67	–	50	50	50	–	50	50	33
*Protoperidinium*	*–*	–	–	33	–	–	–	–	–	–	–	–	–	–	–	–	–
*Pyrocystis*	*–*	–	–	–	–	–	–	–	–	–	–	–	–	–	–	–	–
*Pyrodinium*	*–*	–	–	–	–	–	–	–	–	–	–	–	–	–	–	–	–
*Stephanophycis*	*–*	–	–	–	–	–	–	–	–	–	–	–	–	–	–	–	–
*Thalassionema*	*–*	–	–	–	33	33	50	33	–	50	67	50	83	–	150	50	67
*Thallassiosira*	*–*	–	–	–	–	–	–	–	–	–	–	–	50	–	–	–	–
*Triceratium*	*–*	–	–	–	–	–	–	–	–	–	–	–	–	–	–	–	–
*Rhizoselenia*	50	–	–	–	–	–	50	–	50	33	50	–	50	–	–	50	–
*Unknown*	–	–	–	–	–	–	–	–	–	–	–	–	–	–	–	–	–
**Total density of phytoplankton**	**316**	**116**	**250**	**549**	**299**	**233**	**233**	**200**	**167**	**150**	**267**	**266**	**515**	**33**	**333**	**416**	**100**
**Total no. of genus**	**8**	**3**	**5**	**8**	**6**	**5**	**5**	**4**	**3**	**6**	**5**	**6**	**10**	**1**	**5**	**7**	**2**

**Table 5:** Percentage abundance (%) of the main group of phytoplankton in Pulau Tinggi, Johor.

Month	Stations	Group (%)		
		Diatoms	Dinoflagellates	Others
August	PT1	1.00	–	–
	PT2	1.00	–	–
	PT3	1.00	–	–
	PT4	1.00	–	–
	PT5	1.00	–	–
	PT6	1.00	–	–
	PT7	1.00	–	–
	PT8	1.00	–	–
	PT9	1.00	–	–
	PT10	1.00	–	–

**Table 6:** Percentage abundance (%) of the main group of phytoplankton in Port Dickson, Negeri Sembilan.

Month	Stations	Groups (%)		
		Diatoms	Dinoflagellates	Others
July	S1	96.19	3.81	–
	S2	99.49	0.51	–
	S3	100.00	–	–
	S4	100.00	–	–

September	S5	100.00	–	–
	S6	88.89	11.11	–
	S7	58.82	41.18	–
	S8	72.41	27.59	–
	S9	66.67	33.33	–
	S10	75.00	25.00	–
	S11	75.00	25.00	–
	S12	100.00	–	–
	S13	100.00	–	–
	S14	100.00	–	–

October	S15	88.89	11.11	–
	S16	100.00	–	–
	S17	100.00	–	–
	S18	100.00	–	–
	S19	100.00	–	–
	S20	100.00	–	–
	S21	83.33	16.67	–

November	S22	100.00	–	–
	S23	100.00	–	–
	S24	100.00	–	–
	S25	92.31	7.69	–
	S26	100.00	–	–
	S27	93.33	–	6.67
	S28	100.00	–	–

December	S29	100.00	–	–
	S30	100.00	–	–
	S31	100.00	–	–
	S32	93.33	–	6.67
	S33	100.00	–	–
	S34	100.00	–	–
	S35	100.00	–	–
	S36	100.00	–	–
	S37	100.00	–	–
	S38	60.00	–	40.00

## 4 Experimental Design, Materials and Methods

### 4.1 Sampling activity

All data provided in this article were collected from two locations which were Pulau Tinggi, Johor and Port Dickson, Negeri Sembilan from July 2023 until December 2023 (Fig. 1 and Table 1). Sampling activity in Pulau Tinggi was conducted in August 2023 (station PT1 – PT10). Meanwhile, sampling activities at Port Dickson have been done during the Southwest (SW) monsoon (station S1 - S21) and Northwest (NW) monsoon (station S22–S38). The sampling activities have been done during low to moderate wind speeds (0.11 to 6.07 ms^−1^). For phytoplankton enumeration, the seawater samples from a 1-meter depth were collected using a Niskin bottle and then were concentrated and stored in a 50 ml centrifuge tube. Samples were immediately preserved with Lugol's iodine solution and stored in the dark until microscopic analysis. The ambient photosynthetic active radiation (PAR) was measured using a PAR Smart Sensor (S-LIA-M003, Onset HOBOware).

### 4.2 Species identification and phytoplankton cell counting

The identification of genus was based on morphological characteristics according to the reference of phytoplankton identification [10] and verified with Ocean Data Center (http://oceandatacenter.ucsc.edu/PhytoGallery/phytolist.html) and AlgaeBase (https://www.algaebase.org) Microscopic determination and counting were done using an inverted microscope (Olympus x70) at 10x magnification based on Utermöhl [11]. The samples were determined as far as possible at the genus level.

### 4.3 Chlorophyll-a analysis

Chlorophyll-a concentration was determined by filtering 800–1000 mL of sample onto GF/F (Whatman), extracted with 90 % acetone for 12 h in the dark at 4 °C, centrifuged for 10 min, and then measured with a UV–visible spectrophotometer (UVD-3000, Labomed Inc. USA). Chlorophyll-a concentration was calculated according to [4], substituting the equation from [7].

## Limitations

The data in this article have limitations while collecting the data. Taxonomic identification can be challenging for many phytoplankton taxa since many tiny phytoplankton species cannot be recognised using light microscopy, identification of these organisms is typically done at a coarser taxonomic level.

## Ethics Statement

The authors declare this article's content does not involve human subjects, animal experiments, or any data collected from social media platforms.

## CRediT Author Statement

**Ayu Nabila Khairul Anuar:** Sample analyses, Samples collection, Writing, **Nur Khayyirah Muhammad Zamri**: Sample Analysis, **Muhammad Abid Mohd Yuzaidey:** Samples collection, Chlorophyll-a analysis, **Suriyanti Su Nyun Pau**: Data validation, Supervision, Editing. **Nur Ili Hamizah Mustaffa:** Editing, Conceptualization, Supervision, Funding. All authors read and agree before submission.

## Data Availability

Dataset of taxonomic identification from surface water in the coastal area of Peninsular Malaysia (Original data) (Mendeley Data)
